# Computational modeling of retinal hypoxia and photoreceptor degeneration in patients with age-related macular degeneration

**DOI:** 10.1371/journal.pone.0216215

**Published:** 2019-06-11

**Authors:** Kevin J. McHugh, Dian Li, Jay C. Wang, Leon Kwark, Jessica Loo, Venkata Macha, Sina Farsiu, Leo A. Kim, Magali Saint-Geniez

**Affiliations:** 1 Schepens Eye Research Institute, Massachusetts Eye and Ear, Boston, MA, United States of America; 2 Department of Biomedical Engineering, Boston University, Boston, MA, United States of America; 3 Department of Ophthalmology, Harvard Medical School, Boston, MA, United States of America; 4 Departments of Ophthalmology and Biomedical Engineering, Duke University, Durham, NC, United States of America; 5 Department of Computer Science, Harvard College, Cambridge, MA, United States of America; University of Florida, UNITED STATES

## Abstract

Although drusen have long been acknowledged as a primary hallmark of dry age-related macular degeneration (AMD) their role in the disease remains unclear. We hypothesize that drusen accumulation increases the barrier to metabolite transport ultimately resulting in photoreceptor cell death. To investigate this hypothesis, a computational model was developed to evaluate steady-state oxygen distribution in the retina. Optical coherence tomography images from fifteen AMD patients and six control subjects were segmented and translated into 3D in silico representations of retinal morphology. A finite element model was then used to determine the steady-state oxygen distribution throughout the retina for both generic and patient-specific retinal morphology. Oxygen levels were compared to the change in retinal thickness at a later time point to observe possible correlations. The generic finite element model of oxygen concentration in the retina agreed closely with both experimental measurements from literature and clinical observations, including the minimal pathological drusen size identified by AREDS (64 μm). Modeling oxygen distribution in the outer retina of AMD patients showed a substantially stronger correlation between hypoxia and future retinal thinning (Pearson correlation coefficient, r = 0.2162) than between drusen height and retinal thinning (r = 0.0303) indicating the potential value of this physiology-based approach. This study presents proof-of-concept for the potential utility of finite element modeling in evaluating retinal health and also suggests a potential link between transport and AMD pathogenesis. This strategy may prove useful as a prognostic tool for predicting the clinical risk of AMD progression.

## Introduction

Age-related macular degeneration (AMD) is the leading cause of blindness in people over the age of 50, yet little is known about the underlying pathological processes that ultimately culminate in vision loss.[1] Several early studies of AMD hypothesized that drusen may interfere with sufficient metabolite delivery,[2, 3] while others have demonstrated a substantial decrease in metabolite delivery with aging.[4, 5] Although drusen and Bruch’s membrane (BrM) thickening may more significantly affect the delivery of large metabolites due to decreased macromolecular mobility, oxygen has long been considered the limiting metabolite in the outer retina[6] and has also been identified as a potential factor in retinal disease.[7, 8] Therefore, this study focuses only on the transport of oxygen in the outer retina.

In the healthy outer retina, 85–90% of oxygen is delivered by the choriocapillaris while the remaining 10–15% is obtained from the deep retinal capillary plexus.[9, 10] The majority of oxygen consumed in the outer retina occurs in the photoreceptor inner segment (IS) where densely packed mitochondria produce ATP via aerobic respiration.[11–14] To combat the retina’s high rate of oxygen consumption, the choroid is perfused at a rate that minimizes the drop in blood oxygen concentration along the vascular plexus to just 1% between arteriole and venule.[15, 16] Drusen and Bruch’s membrane (BrM) thickening associated with both age and disease increase the barrier between the choriocapillaris[17, 18] and the highly metabolically active outer retina resulting in critically reduced metabolite delivery.[19] Although hemoglobin and neuroglobin can improve the oxygen concentration in the blood and retinal pigment epithelium (RPE), respectively,[20] only soluble oxygen is accessible to photoreceptors. Consequently, the rate limiting step to oxygen delivery in AMD is likely to be extravascular transport via diffusion rather than choroidal ischemia.[18]

The current approach used to diagnose and stage dry AMD is based on the number and width of drusen observed on fundus photographs, criteria established by the Age-Related Eye Disease Study (AREDS).[21] Although useful for assessing the current stage of AMD, AREDS classifications are poor predictors of future retinal degeneration. Five years after diagnosis, only 43% of patients diagnosed with the most severe form of intermediate AMD progress to an advanced form of the disease.[22] Morphological features including drusen-RPE thickness have been shown to correlate with deterioration of vision,[23] and drusen volume, fundus autofluorescence abnormalities, and optical coherence tomography (OCT)-based scoring systems have also improved on the prediction of progression of AMD.[24–26] However, there is still a clinical need to develop a prospective method that more accurately predicts AMD progression. We hypothesize that drusen and Bruch’s membrane thickening can impede oxygen transport via increased diffusion distance and possibly slower transport resulting in hypoxia at the photoreceptor IS that can lead, over time, to retinal degeneration. In this study, OCT images were used to reconstruct morphology in silico and model local oxygen concentration in a cohort of AMD patients, which has been recently suggested as a potentially promising approach.[27] This technique demonstrates a significant correlation between retinal hypoxia and future retinal thinning, which may be clinically useful as a predictor of disease progression in AMD.

## Materials and methods

### Summary

This approach used semi-automated segmentation of OCT images to recreate outer retinal geometry in silico. Oxygen concentration across the outer retina was then evaluated using finite element analysis to identify regions of hypoxia that are at risk for future retinal degeneration. In this study, longitudinal patient data was used to compare predicted retinal hypoxia with actual downstream photoreceptor layer thinning in patients approximately one year later.

### Finite element modeling of retinal oxygen distribution in a generic retina

**Fig 1:**
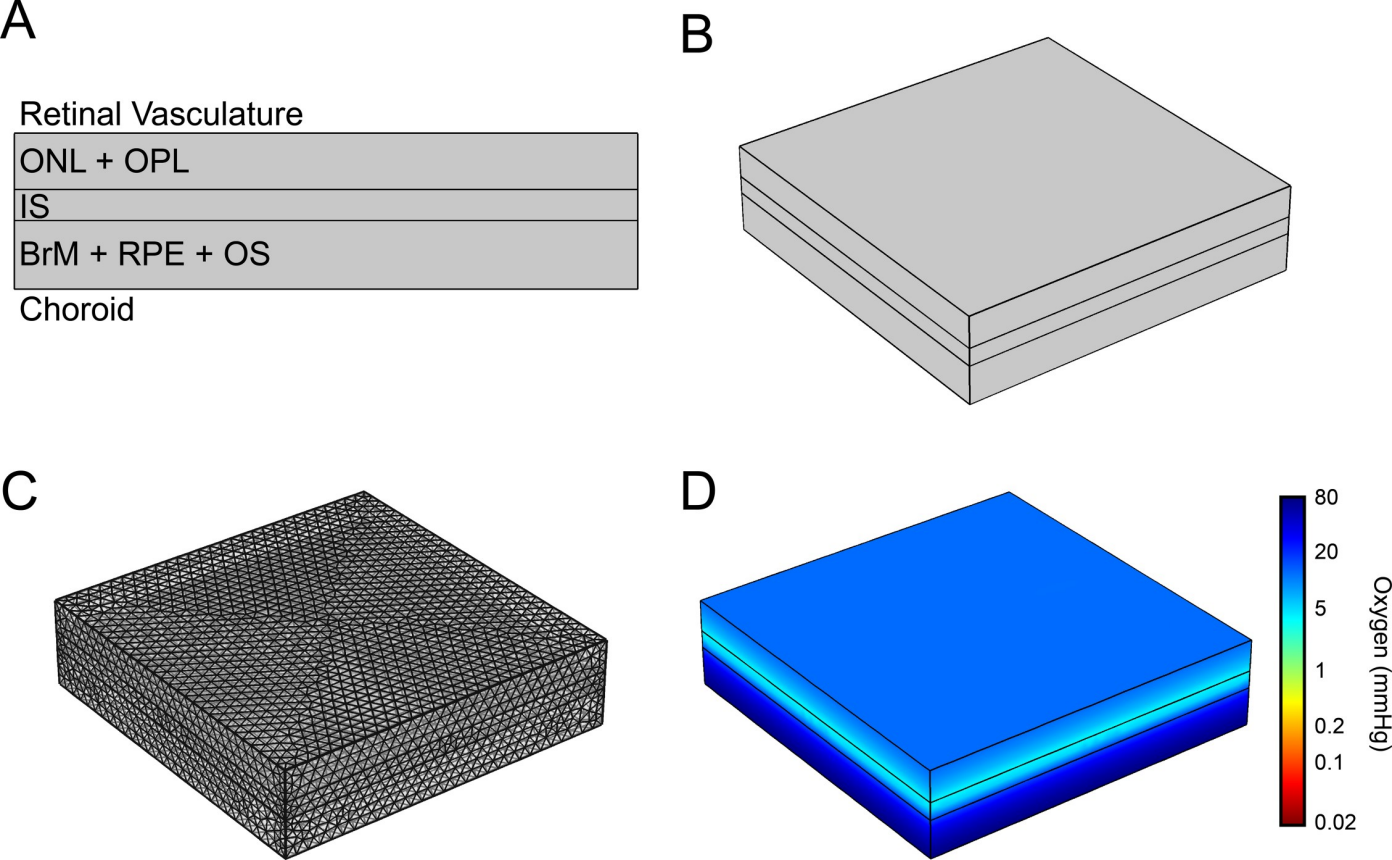
In silico evaluation of oxygen in a healthy retina. (A) Structure of the laminar generic retina including choroid, Bruch’s membrane (BrM), retinal pigment epithelium (RPE), photoreceptor outer segment (OS), inner segment (IS), outer nuclear layer (ONL), outer plexiform layer (OPL), and retinal vasculature. (B) Three-dimensional representation of retinal geometry in COMSOL. (C) Meshed geometry in which intersections are points calculated in the finite element model based on connecting vertices, and (D) after the evaluation of the partial pressure of oxygen across the retina at steady state.

COMSOL Multiphysics (Burlington, MA) was used to evaluate steady state oxygen concentration in the dark-adapted retina determined using 3D representations of outer retinal morphology. This approach employed the Transport of Diluted Species physics engine to study oxygen transport and consumption in the retina. Studies used anatomical and physiological parameters based on values previously reported in the literature (Table 1). A fixed oxygen concentration was applied at the basal side of the BrM domain and constant oxygen influx was set for the apical surface of the OPL to represent the hyperperfused choriocapillaris and deep retinal vasculature, respectively. Oxygen consumption was set to occur only within the IS layer where the vast majority of retinal oxygen is used by photoreceptor mitochondria for aerobic respiration while other areas were set to zero.[19, 28] The diffusion coefficient of all domains was set equal to the values identified experimentally in cats.[29] Finite element analysis was then performed to determine the steady state oxygen distribution throughout the retina (Fig 1). The critical assumptions used in this model were: (1) Oxygen consumption occurs only within the photoreceptor IS and the rate of consumption is proportional to the volume occupied by this layer.[30–32] (2) Oxygen transport within retinal tissue obeys Fick's laws of diffusion.[29, 33] (3) The choriocapillaris is a homogeneous boundary at a fixed oxygen concentration corresponding to highly oxygenated blood and a high perfusion rate.[15, 33, 34] (4) The deep retinal vasculature provides constant flux at the inner boundary of the OPL due to its ability to regulate flow.[31] (5) Retinal curvature is negligible at the length scale of oxygen diffusion.[30, 35]

**Table 1:** Finite element modeling parameters.

Parameter	Value
Oxygen diffusion coefficient in retinal tissue^29^	1.97 x 10^−9^ m^2^/s
Oxygen consumption rate within IS (dark-adapted)^33^	0.148 mol/m^3^/s
Soluble oxygen concentration (pO_2_) at the choroid^15,33, 34^	0.1107 mol/m^3^ (80 mmHg)
Soluble oxygen flux from retinal vasculature^a^	5.05 x 10^−7^ m^2^/s
Combined BrM, RPE, and OS height^28^	55 μm
IS height^14,32^	25 μm
Combined ONL and OPL height^14^	45 μm

^a^Note: Determined empirically due to lack of published data. IS = inner segment, BrM = Bruch’s membrane, RPE = retinal pigment epithelium, OS = outer segment, ONL = outer nuclear layer, OPL = outer plexiform layer.

Layers were assembled in the model using the block tool in which retinal layers were treated as rectangular slabs. A value for the flux of oxygen from the deep retinal vasculature was unavailable in literature; therefore it was empirically determined to mimic the profile of oxygen concentration through the depth of the retina measured experimentally in the feline eye.[36]

In a scenario meant to approximate early dry AMD, one hemispherical "druse" was introduced underneath the RPE causing layers above the druse to be displaced accordingly using the sphere and Boolean tools. The diameter of this hemispherical druse was then varied to observe the effects of size on retinal oxygen concentration using extra fine meshing. The effect of a wide, but thin feature mimicking soft drusen was also studied to observe changes in steady state oxygen concentration using the cylinder geometry, Boolean tools, and fine meshing to reduce computation power. These short, yet wide cylinders were placed perpendicular to the retinal plane such that the height corresponded to druse height and the radius represented the lateral expanse of the druse.

### Patient data acquisition and anonymization

The use and anonymization of OCT images were performed in a HIPAA-compliant manner. Institutional Review Board (IRB)/Ethics Committee approval was obtained from Massachusetts Eye and Ear (MEE) Human Studies Committee (IRB #427282–1). This work adhered to the tenets of the Declaration of Helsinki.

OCT images in the existing MEE database were analyzed to identify patients with both retinal pathology characteristic of dry AMD (sub-macular drusen) and OCT images from multiple time points. In total, thirty OCT volume scans from 15 patients with intermediate AMD and twelve OCT volume scans from 6 control subjects without AMD were selected and exported as E2E files. If both of a patient’s eyes satisfied the criteria for inclusion, one was randomly selected for inclusion in the study. All potentially-relevant patient information (gender, age, date) and clinical information (visual acuity, stage of AMD, treatment, and conversion to wet AMD) was stored in a password-protected file and all identifying information was removed from OCT images.

### Segmentation of optical coherence tomography images

B-scans were automatically segmented as a series using software previously described[37] and then each B-scan was carefully reviewed and corrected manually by a grader if required resulting in 8 layers: RPE, drusen, and BrM (RPE-drusen-BrM), OS, IS, outer nuclear layer (ONL), outer plexiform layer (OPL), inner nuclear layer (INL), inner plexiform layer (IPL) and ganglion cell layer (GCL). The corresponding fundus images were then used to integrate multiple segments into three-dimensional spatial coordinates in MATLAB (Mathworks, Natick, MA). The curvature of the outermost retina was used as the baseline for retinal thickness to simplify modeling geometries. To ensure accurate alignment between the in silico retinal morphologies at the early and late time point, RPE-drusen-BrM layers were first manually aligned. One layer was then rotated and translated in the X and Y directions to identify the optimal alignment between layers. The orientation that resulted in the highest Pearson’s correlation coefficient between the RPE-druse-BrM height at the first and second image collation time points was used for subsequent analysis. Retinal geometries were then cropped to 3000 x 3000 x 400 μm in X, Y, and Z centered at the deepest foveal spot in order to generate a COMSOL-compatible geometry for input purposes. Lastly, IS thickness could not be reliably traced due to insufficient OCT resolution and therefore was recalculated based on the relative thickness of the overlying ONL and OPL. The IS below the thickest ONL and OPL was set to 25 μm-thick and the IS thickness at all other locations was set to a linear fraction of that thickness based on overlying ONL and OPL thickness. These coordinates were then passed into Global Mapper 14 (Blue Marble Geographics, Hallowell, ME) and exported in digital elevation model (.dem) format to create a patient-specific topographical map of the retina that could be imported into COMSOL Multiphysics. Thinning of the ONL and OPL over time was determined by comparing the thickness of the segmented layers from the baseline (first) and second OCT scans. The segmented geometries from the two time points were cross-registered as described above and used to calculate the difference between the combined thickness of the ONL and OPL over time.

### Patient-specific finite element modeling

Patient-specific finite element modeling studies used custom geometries obtained from OCT segmentation as previously described. Topographical maps of six layers corresponding to the boundaries between outer retinal layers were imported into COMSOL as parametric surfaces and used to create a solid continuous structure with multiple domains. The domains of interest were: (1) BrM, (2) drusen, (3) RPE and OS, (4) IS, (5) ONL, and (6) OPL. For simulation and evaluation purposes, the ONL and OPL were combined into one layer since no differences in properties would be expected. The assumptions and physiological parameters applied in this model were the same as those used for the generic retinal model. In order to avoid potential oxygen concentrations below zero, which is not physically possible and therefore could invalidate the resulting data, we employed a step function where concentration was set to zero if it would otherwise have been negative.

### Spatial correlation

Steady-state oxygen values were down-sampled to 150x150x20 (XYZ) matrices and exported to Microsoft Excel for subsequent quantitative analysis. Spatial correlation studies were performed by grouping together the fifteen eyes showing clinical signs of AMD, six control eyes, or all twenty-one eyes. The 20 Z-values at each XY coordinate were scanned to identify the minimum oxygen concentration at that point on a two-dimensional map, which resulted in 22,500 data points from each eye. To investigate the effect of drusen on baseline retinal status, the combined thickness of the ONL and OPL was compared to the thickness of the directly underlying RPE-drusen-BrM. The combined ONL and OPL thickness rather than the ONL thickness alone was used to avoid potential OCT imaging artifacts associated with imaging angle and Henle fiber visibility.[38] A Pearson correlation coefficient (r) was calculated to evaluate the overall spatial correlation between RPE-drusen-BrM thickness and changes in ONL and OPL thickness over time. To assess the co-localization of retinal hypoxia and future thinning of the ONL and OPL, the minimum oxygen concentration at each XY point (always located within the oxygen-consuming IS layer) was compared to the decrease in combined ONL and OPL thickness between the two imaging time points. A Pearson correlation coefficient was again calculated for the overall spatial correlation between minimum oxygen concentration and adjacent ONL and OPL thinning for all eyes.

## Results

### Generic single druse finite element modeling

**Fig 2:**
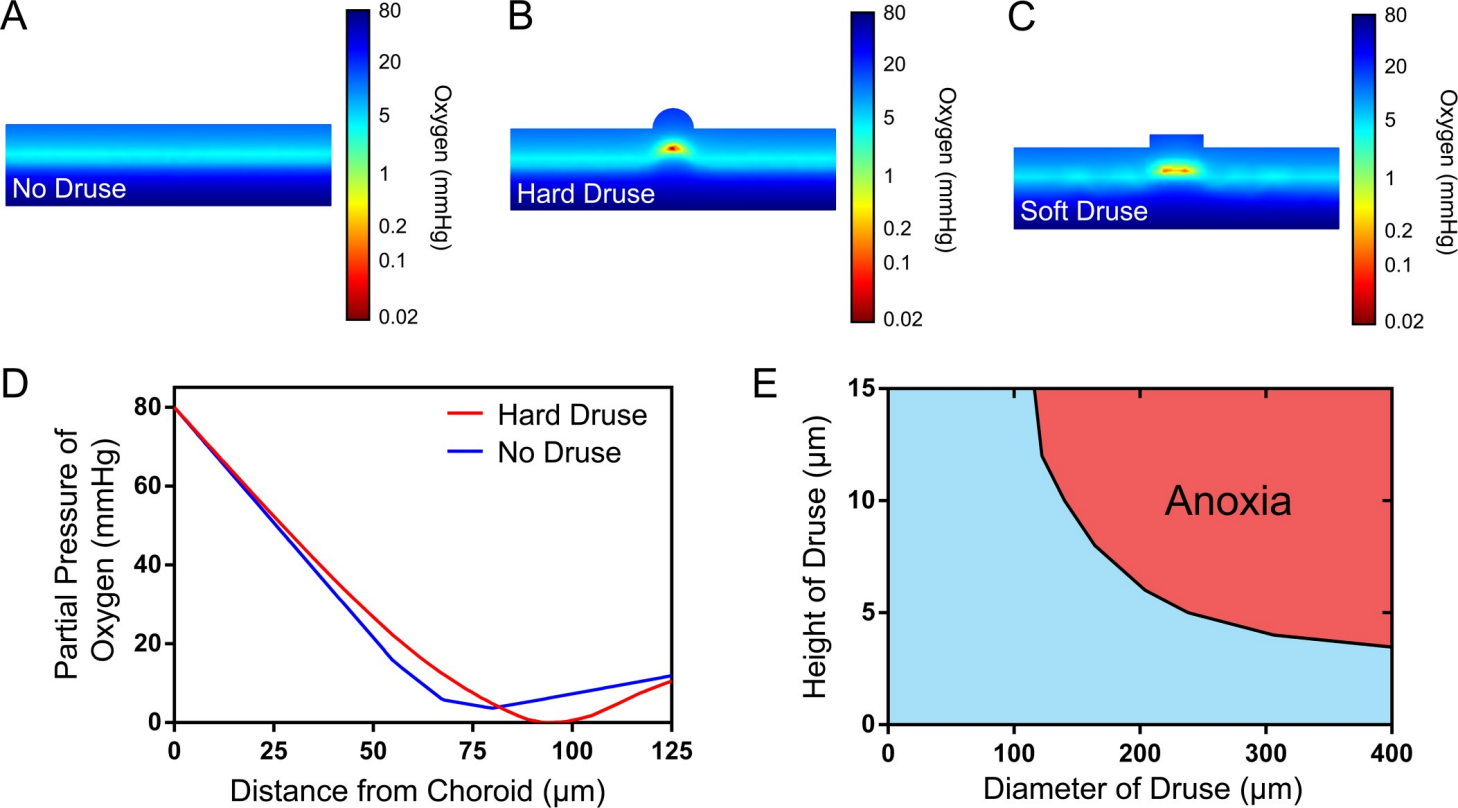
Oxygen distribution across the diseased retina. Cross-sectional view through the center of (A) a healthy retina without pathology, (B) a diseased retina with a hemispherical “hard” druse 64 μm in diameter that displaces the immediately overlying retinal layers, and (C) a diseased retina with a cylindrical “soft” druse that is 82 μm in diameter and 20 μm in height. The drusen sizes shown are just large enough to pose a transport barrier resulting in a small region of anoxia (i.e. partial pressure of oxygen equal to zero) immediately overlying the center of the druse, as show in red. (D) Computational data showing a low, but non-zero partial pressure of oxygen in the healthy retina and anoxic retina in the presence of a hemispherical druse 64 μm in diameter. In both cases the minimum oxygen levels are observed in the inner segment layer, though the druse causes this layer to be vertically displaced resulting in the rightward shift observed here. (E) Drusen such as the soft drusen modeled here as cylinders are capable of inducing regions of anoxia as a function of both their height and diameter due to direct (increased diffusion distance) and indirect (compensatory lateral diffusion), respectively.

A computational model depicting steady state oxygen concentration throughout the outer retina was constructed to match anatomical and physiological data reported in the literature (Fig 1). The partial pressure of oxygen across the model was lowest within the IS layer, as expected, reaching a minimum of 4 mmHg. The system was then perturbed by the addition of a feature mimicking a druse immediately adjacent to the BrM, which deformed subsequent layers above the retina and altered oxygen distribution (Fig 2A–2C). Increasing the size of the hemispherical feature mimicking the morphology of a hard druse resulted in lower oxygen levels throughout the retina, reaching near complete anoxia in the IS directly above the center of the druse at a diameter of 64 μm (Fig 2D). Anoxia could also be achieved using cylindrical geometries with lower aspect ratios simulating soft drusen (Fig 2C), though these features required much larger lateral dimensions (Fig 2E). For example, drusen that were 5, 10, 15, and 20 μm thick began to induce anoxia at diameters larger than 238, 140, 116, and 82 μm, respectively. Interestingly, morphological features simulating drusen that were less than 4 μm in height were unable to induce anoxia regardless of lateral dimensions.

### Patient demographics

The clinical and demographic characteristics of the patients with intermediate non-exudative AMD and controls are summarized in Tables 2 and 3. The average age of patients with AMD at the time of initial OCT imaging utilized for segmentation and finite element analysis was 75 ± 9 years and for controls was 71 ± 6 years. Follow-up OCT images used to assess changes in retinal thickness over time were obtained after an average of 13 ± 6 months for AMD patients and 10 ± 1 month for controls. At the end of the clinical follow-up period, 6 of 15 patients in the AMD group converted from dry to wet macular degeneration in the eye that was used for modeling and analysis after an average of 48 ± 25 months.

**Table 2:** Demographic and clinical characteristics of patients with intermediate AMD.

Age* (years)	Sex	Race	Eye used	Smoking status	FH AMD	Visual acuity*	Time between OCT images (months)	Total follow up time (months)	Status of fellow eye	Conversion to wet AMD?	Time to wet AMD conversion (months)	Conversion of fellow eye to wet AMD?
**85**	F	W	OS	Non	No	20/32	13	72	Dry	Yes	59	Yes
**73**	F	W	OS	Non	Yes	20/25	13	69	Dry	Yes	44	Yes
**65**	F	W	OD	Non	Yes	20/32	20	86	Dry	Yes	68	Yes
**73**	M	W	OS	Non	No	20/63	6	56	Dry	No	-	No
**82**	F	W	OS	Former	No	20/30	10	50	Wet	Yes	15	-
**78**	F	W	OD	Non	No	20/20	10	49	Wet	No	-	-
**84**	F	W	OS	Former	Yes	20/30	10	49	Wet	Yes	22	-
**65**	F	W	OS	Former	No	20/30	33	48	Dry	No	-	No
**78**	F	W	OS	Non	No	20/25	11	85	Wet	Yes	78	-
**84**	M	W	OS	Former	No	20/30	11	50	Wet	No	-	-
**92**	M	W	OS	Non	No	20/50	10	34	Wet	No	-	-
**69**	F	W	OD	Non	Yes	20/25	10	31	Wet	No	-	-
**65**	F	W	OD	Former	No	20/32	10	40	Dry	No	-	-
**60**	F	W	OD	Non	No	20/20	16	43	Dry	No	-	-
**67**	F	W	OD	Former	Yes	20/20	11	36	Dry	No	-	-

*At inclusion of study. F = female, M = male, W = white, OS = left eye, OD = right eye, FH = family history, AMD = age-related macular degeneration, OCT = optical coherence tomography.

**Table 3:** Demographic and clinical characteristics of control subjects.

Age* (years)	Sex	Race	Eye used	Smoking status	FH AMD	Visual acuity*	Time between OCT images (months)	Clinical changes
**63**	F	W	OD	Non	No	20/20	9	None
**64**	M	Unknown	OS	Non	No	20/20	10	None
**77**	M	Unknown	OD	Non	No	20/25	11	None
**76**	F	W	OD	Former	No	20/25	10	None
**75**	M	Unknown	OD	Former	No	20/20	10	None
**73**	F	W	OD	Non	No	20/25	9	None

*At inclusion. F = female, M = male, W = white, OS = left eye, OD = right eye, FH = family history, AMD = age-related macular degeneration; OCT = optical coherence tomography.

### Patient-specific finite element modeling

**Fig 3:**
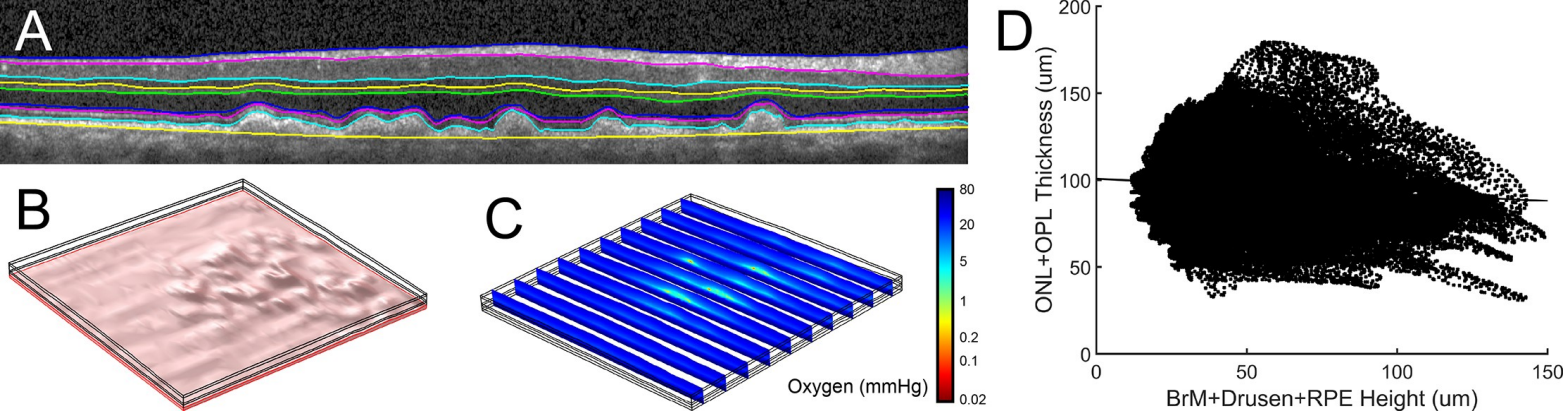
Patient-specific retinal mapping and finite element modeling. (A) Semi-automated segmentation of the retina was performed by evaluating sequential OCT B-scans. (B) Three-dimensional drusen morphology shown in a patient’s retina using co-registration of B-scans with fundus photography. (C) Ten evenly-spaced cross-sections showing the partial pressure of oxygen throughout a patient’s retina including regions of mild hypoxia near the fovea.

**Fig 4:**
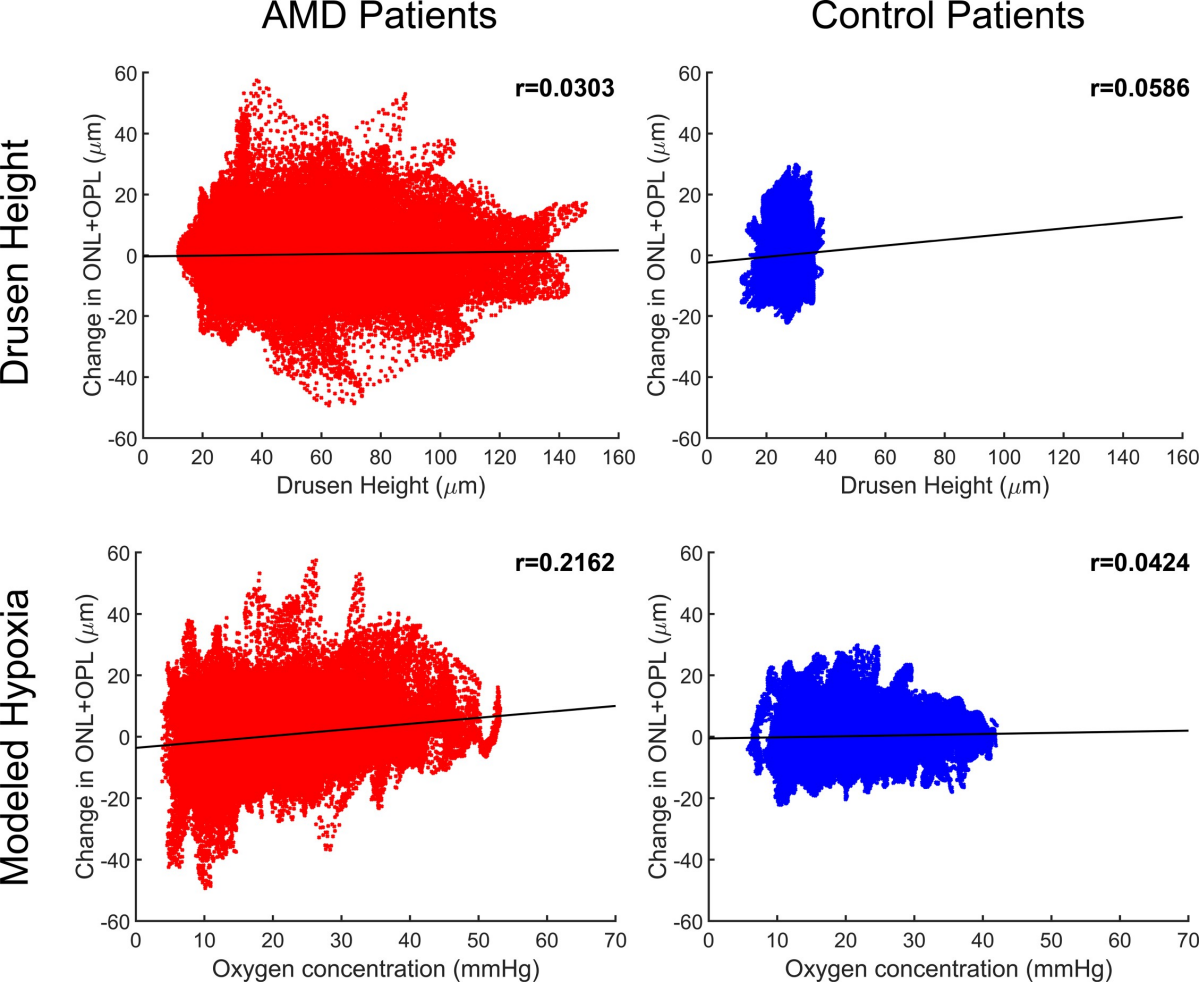
Correlation between future retinal thinning and drusen height (row 1) or modeled steady-state oxygen levels (row 2) at the same retinal location. The y-axis represents the change in ONL and OPL thickness between the initial and follow-up time point with negative values representing thinning of the ONL + OPL. Column 1 shows data from 15 AMD patients and Column 2 shows data from 6 control subjects (n = 22,500 data points per patient from a 150 x 150 value spatial map). The correlation between drusen height (i.e. BrM-drusen-RPE thickness) and future retinal thinning is positive–opposite of what might be expected—but rather weak for both groups (r = 0.0303 for AMD, r = 0.0652 for controls). The correlation between steady-state oxygen concentration and retinal thinning was much stronger for AMD patients (r = 0.2162) suggesting that the model may be able to better predict future retinal degeneration than drusen dimensions because of its ability to incorporate additional factors such as drusen width and previous retinal thinning.

Retinal hypoxia was determined using finite element modeling based on OCT data from the initial imaging time point (Fig 3) and compared to the change in retinal layer thickness approximately one year later. Spatial correlation analysis of drusen height and retinal thinning was used to determine if physiological modeling provided a greater degree of insight into future patient outcomes than simple automated anatomical measurements. Although drusen height was expected to have a negative correlation with a change in combined ONL and OPL thickness (i.e. thicker drusen would be associated with greater degradation), the opposite trend was observed (Fig 4). The Pearson correlation coefficient for drusen height and future thinning of the ONL and OPL was weakly positive for AMD patients (r = 0.0303), control subjects (r = 0.0652), and the combined populations (r = 0.0288). In fact, thicker drusen were actually weakly associated with less ONL and OPL thinning. Alternatively, the Pearson correlation coefficient for oxygen concentration and future ONL and OPL thinning was positive. While the relationship between these parameters was relatively weak for control subjects (r = 0.0349), a substantially stronger correlation was observed for AMD patients (r = 0.2162). Due to the large number of data sets in each population, both correlations were highly significant (p<0.0001), though data points are not fully independent.

**Fig 5:**
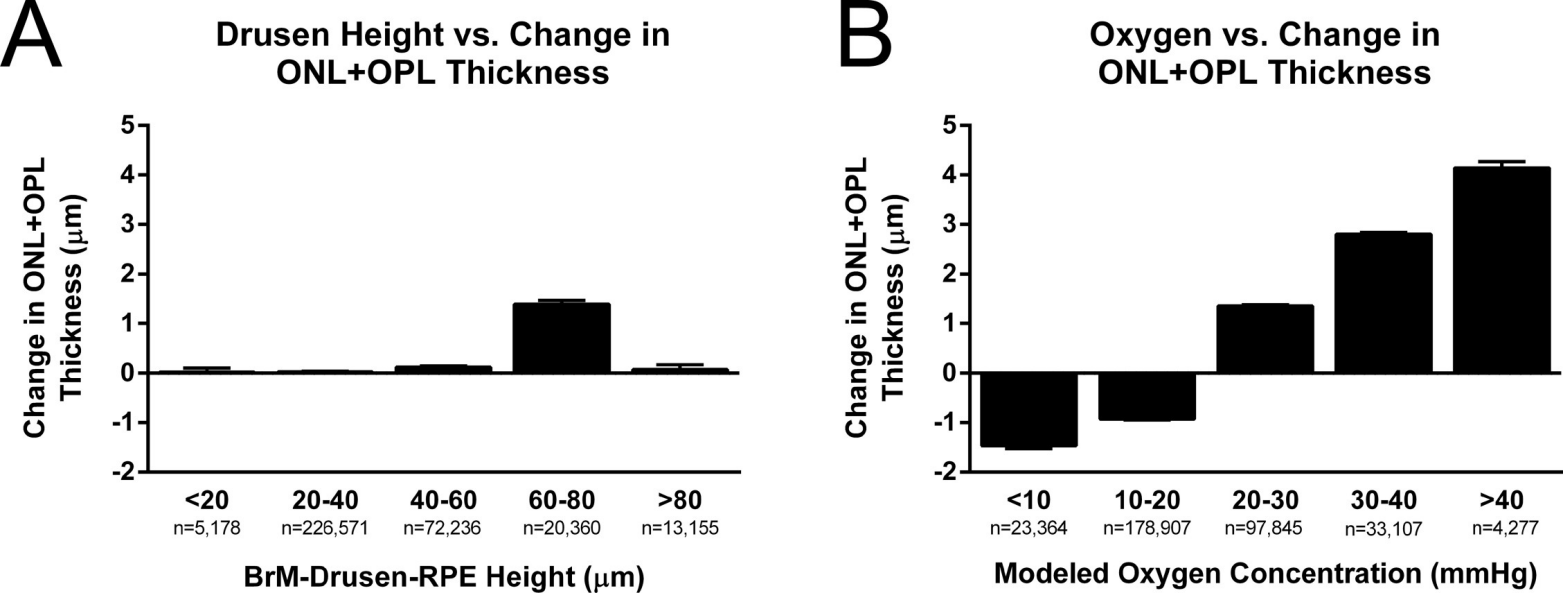
Histograms showing the average change in retinal thickness. Comparison of ONL + OPL thickness to (A) drusen height (i.e. BrM-drusen-RPE thickness) and (B) minimum at the same corresponding location for AMD patients between the two time points collected. There is no obvious trend in the relationship between drusen height and future retinal thinning; however, a more obvious trend is seen between predicted oxygen concentration and retinal thinning. Error bars indicate standard error of the mean.

The relationships observed for AMD subjects in Fig 4 became more obvious when displayed as histograms. Minimal association was observed between drusen height and future retinal thinning with four of the five histogram bins very close to zero while the last demonstrated a change of only 1.4 μm on average (Fig 5A). In contrast, there was a clear trend between predicted oxygen concentration and a decrease in ONL and OPL thickness at the second imaging time point (Fig 5B). This range of average outcomes was also much more diverse. Areas predicted to experience hypoxia (<10 mmHg) thinned by an average of 1.5 μm whereas areas regions predicted to be well-oxygenated (>40 mmHg) increased in thickness by an average of 4.1 μm. Further, all oxygen concentration bins yielded significant differences in average ONL and OPL thinning (p<0.0001) as determined using a one-way ANOVA with Tukey’s multiple comparisons test.

## Discussion

The generic model of retinal oxygen equilibrium identified a hemispherical druse with a diameter of 64 μm as a key threshold above which anoxia begins to occur. This value is in direct agreement with the smallest hard druse that is treated as pathological in the AREDS classification system.[22] The finding that anoxia could not be induced by soft drusen less than 4 μm in height regardless of lateral dimension also agrees with experimental data collected by Linsenmeier and colleagues in cats and non-human primates.[9, 10] Those studies show that the healthy outer retina has a small excess of oxygen—even in the dark-adapted retina—so minor increases in the diffusion distance could be tolerated. Together, these findings suggest that excess oxygen in the normal outer retina serves as a buffer that confers protection against small, otherwise-deleterious physiological or morphological changes (e.g. BrM thickening, small drusen accumulation, reduced blood perfusion) prior to the onset of overt pathology.

Although oxygen diffusion from the choroidal vasculature to photoreceptors in the healthy retina is perpendicular to the retinal axes, drusen can decrease oxygen levels directly above each druse leading to the lateral diffusion of oxygen down a concentration gradient. This lateral transport phenomenon can help to explain why hemispherical drusen that are quite tall (e.g. 30 μm) can fail to induce severe hypoxia if they are relatively narrow while thinner (e.g. 5 μm) drusen can induce complete anoxia if they are large enough in the lateral dimensions. This finding is also consistent with clinical observations from the Copenhagen study, which showed that soft drusen are more highly associated with progression to central geographic atrophy or choroidal neovascularization (CNV) than hard drusen.[39] Although it may seem disadvantageous for the eye to evolve with such a small tolerance for BrM thickening and drusen deposition, the disadvantage of late-onset pathology may be outweighed by the advantage of minimizing oxidative stress by reducing excess oxygen in a region that is consistently exposed to light with potential toxicity.[7]

Patient-specific analysis provided additional information about the potential importance of oxygen transport in the diseased retina. Because of the limited availability of data that fit our inclusion criteria (multiple visits with OCT images, disease status, etc), there may have been racial differences between the control and AMD groups; however, this would not be expected to affect our model because rates of outer retinal thinning have not been shown to be affected by race or ethnicity. In addition, the finding that 6 of 15 patients in the AMD group converted from dry to wet macular degeneration after approximately four years is similar to previously published data, suggesting that our patient pool is representative of the overall population in this group.[22]

Finite element analysis was used to evaluate both the hypothesis that (1) drusen height would cause the photoreceptors directly above to degenerate over time and that (2) steady-state oxygen levels in the retina would correlate well with future retinal thinning. The data presented here does not support the first hypothesis and actually suggests that drusen height may be negatively correlated with future retinal thinning. This paradoxical observation may be explained by hypoxia-induced photoreceptor cell death, which would lower oxygen consumption requirements and result in a new steady-state equilibrium. This postulate is supported by previous imaging studies showing that photoreceptor layer thinning is present in areas of the macula affected by drusen.[40] However, because there is no longitudinal data in these studies, it is not possible to determine whether photoreceptor layer degeneration was in progress or had already achieved a new equilibrium. Therefore, it was important that our study include longitudinal morphology to enable the evaluation of downstream changes in the retina. This type of prospective data is also more clinically valuable than simply assessing current retinal status.

Alternatively, our second hypothesis that steady-state oxygen levels would correlate well with future retinal thinning was well supported by the data. By analyzing images collected at multiple time points, we found that thinning of the combined ONL and OPL was better correlated with model-predicted hypoxia than drusen height suggesting that oxygen diffusion limitations may play a more significant role than physical deformation in photoreceptor layer thinning. In some cases, the predicted retinal oxygen concentration was higher than might be initially expected, which could be a consequence of previous retinal degeneration reducing the oxygen requirements of a portion of the retina. This type of over-correction may be possible due to the substantial lag time between the onset of hypoxia and photoreceptor cell death or be a result of secondary pathology resulting from hypoxia such as anaerobic acidification of the retina or immune cell damage. These results agree with evidence in the literature that retinal hypoxia plays a role in AMD pathogenesis. One key hallmark of geographic atrophy is vascular dropout of the choriocapillaris, which would reduce oxygen delivery, and has also been shown to negatively impact visual acuity.[41, 42] It is also likely that CNV is a physiological response to hypoxia that is deleterious when it occurs in the retina.[43] Further, while hypoxia has been shown to increase inner retinal circulation in rats,[44] it is a known stimulus for the production of hypoxia-inducible factor (HIF) and vascular endothelial growth factor (VEGF),[19, 45] which are key drivers of CNV.

As the first study of its type, this model has several limitations. First, the physical and transport parameters of the model may not be representative of a human patient as many were collected from other species. Further, as the oxygen diffusion coefficients for soft and hard drusen are not known, our model does not distinguish between the two. The model also does not consider potential changes to vasculature morphology or transport, which could play a major role in human disease. Finally, in this paper we have limited our analysis to oxygen transport; however, enhanced barriers to diffusion could negatively affect the delivery of other essential molecules and metabolites into or out of the outer retina.

## Conclusions

This study provides proof-of-concept for the use of computational modeling as a method to predict AMD progression. Further, to our knowledge, this is the first study of its kind to evaluate metabolite transport in retinal tissue using OCT images as a patient-specific input. The data also suggests that this physiology-based approach may be superior to the use of simple morphological metrics, such as drusen height, because it reflects both 3D drusen morphology as well as the dynamic nature of retinal degeneration. Taken together, the results from the generic and patient-specific models suggest that reduced oxygen transport may play a key role in AMD disease progression.

Despite using just a single form of patient data, this approach was able to demonstrate a correlation between modeled oxygen levels and future retinal thinning. While there is certainly the possibility of incorporating additional forms of patient data into the model, such as vascular morphology and blood flow measurements, the value of each input should be weighed against the cost and difficulty of obtaining that data. To date, no models bridging blood flow and retinal oxygen exist.[46] For example, while others have demonstrated the feasibility of non-invasive oxygen monitoring within the retinal vasculature using hyperspectral imaging, this technique has not been applied to patients with AMD and does not measure oxygen concentration within non-vascular retinal tissue.[47] One benefit of using OCT data is that it is already commonly collected during clinical visits and therefore would not add any time or inconvenience. However, as the first report of its type, there are still many aspects of this model that could be optimized to improve predictive capabilities including increasing patient enrollment, improving OCT resolution and co-registration, and refining modeling parameters. The long-term goal of this approach is to develop a robust model that can accurately predict future retinal degeneration at the individual patient level. Ultimately, this data could also help to identify potential interventions that ameliorate transport issues such as oxygen supplementation, which has been shown in small clinical studies to improve vision in reduced-transport pathologies including AMD,[48] diabetic macular edema,[49] and cystoid macular edema.[50]
